# What can we do to improve the peer review system? – A short survey of *Food Technology and Biotechnology* peer reviewers' experience

**Published:** 2019-12

**Authors:** Iva Grabarić-Andonovski, Zrinka Pongrac-Habdija, Vladimir Mrša

**Affiliations:** Food Technology and Biotechnology journal, Faculty of Food Technology and Biotechnology, University of Zagreb, Pierottijeva 6, 10000 Zagreb, Croatia

A major part of the scientific editors’ job is finding suitable reviewers for the articles submitted to the journal. As most editors know, this is the most challenging and time-consuming task. The peer review was introduced in 1831 by professor William Whewell from Cambridge University, who convinced the Royal Society in London, the publisher of the first scientific journal Philosophical Transactions (established in 1665), to commission reports on manuscripts submitted to the journal (*1*). Professor Whewell's first objective was to increase the public visibility of science, and only later in 1892 the idea that editors and referees ought to ensure the integrity of the scientific literature began to take hold.

Peer review is a term borrowed from the procedures that government agencies use to decide who would receive research grants (*1*). According to the largest survey of grant peer review, the researchers spend around 10 days each year reviewing academics' funding proposal (*2*). From 4700 researchers, 78% think that peer review is still the best way to allocate research funds, although half of them think that it lacks transparency and objectivity. For each grant proposal, the funder has to contact in average three reviewers to get one who will review the proposal. More than half of the researchers stated that they would more likely accept the request if funders recognized their efforts, either by a thank you note, or by being acknowledged on the funder's reviewer list.

Global State of Peer Review report by Publons published in 2018 surveyed more than 11 000 researchers and concluded that the reviewer fatigue is setting in and that the journal editors have to invite more reviewers in order to receive the review (*3*). The invitation rate increased from 1.9 invitations in 2013 to 2.4 in 2017 for each review, which is why 75% of journal editors say the hardest part of their job is finding willing reviewers. Furthermore, 10% of reviewers, mostly from the USA, China, UK, Japan, Germany, Canada and Australia are responsible for 50% of reviews. Although 41% respondents see peer review as part of their job, 42% of them decline review requests because they are too busy. A large percentage (71%) decline the request for the review because the article is outside their area of expertise. This fact questions the process of selecting reviewers by editors, as well.

McNair *et al.* (*4*) conducted a survey in 2019 among 1203 academics in 10 countries (Germany, Netherlands, Canada, Switzerland, UK, USA, Australia, Japan, Singapore, and China and Hong Kong) and concluded that the average number of accepted peer review tasks per month is 3.5, with the lowest number in Germany (2.7) and the highest in China and Hong Kong (4.9). The percentage of academics who were assisted by early-career researchers during their tasks as a peer reviewer was from 27% (China and Hong Kong) to 67% (UK). More than half of the researchers (55%) have not offered the peer review under their supervision to young researchers, although Committee on Publication Ethics (COPE) guidelines encourage it as part of training and mentoring.

The lack of distribution of peer review invitations is a major problem, but the second one is the acknowledgement of the reviewers. Publons was launched in 2013 in order to provide a platform for tracking publications, citation metrics, peer review and journal editing work (*5*). It now collects data from over 2,000,000 researchers, allowing them to claim credit for reviews and also has an annual peer review award. Services Publons Reviewer Recognition and Reviewer Connect are also provided for the journals, but the fees are quite high for a small journal such as *Food Technology and Biotechnology*.

Reviewer Credits is another platform (free for use by both reviewers and editors) endorsed by University of Milan-Bicocca, which allows scientists to register reviews and conference talks and offers peer review certification (*6*). Reviewers for *Food Technology and Biotechnology* are offered to register their reviews with Reviewer Credits after the completion of their task. They are also acknowledged in the list of reviewers, which is published in the last issue of each year and available online at the journal's webpage.

Besides acknowledgement and certification, some journals are offering financial rewards to the reviewers, publishing fee reduction/waivers or free access to databases such as Science Direct or Scopus (*7*, *8*). Financial reward does not seem to be the main motivation for the researchers. Most of the winners of Publons annual peer review award feel that their duty is to help science and 80% reviewers agree that peer reviewing helps them to enhance their knowledge (*9*). Although reviewers find the invitation for a review as a recognition of their expertise, they would certainly appreciate some kind of acknowledgement or credit during promotion or tenure consideration.

*Food Technology and Biotechnology* journal has three levels of manuscript evaluation, first by the Editor-in-Chief, then by Field editors and finally by reviewers. From 408 papers submitted in 2019, 281 were rejected without reviewing due to poor quality, lack of novelty, or their topic was out of the journal's scope. Of 127 articles that were considered adequate for the reviewing process, 37 were rejected by the Field editor and the rest (90 papers) were sent to the reviewers. The average reviewer's invitation rate was 5.6 and the average time for receiving reviewers' opinions was 110.7 days. This confirms the premise that the editor's task to obtain good quality reviews is not easy at all.

In order to investigate the possible reasons for such high invitation rate (*i.e.* low response from the reviewers), we have conducted a short survey among the reviewers using SurveyMonkey. We sent it to 2000 reviewers from our reviewer database, and collected responses from 5 to 29 December 2019. In that period, we received only 100 responses, most probably due to holiday season. The questions in the survey were the following: did they receive the invitation to review the paper, was the time to complete the review (3 weeks) long enough, is the reviewer's form clearly written and with enough details, what were the reasons for rejecting the reviewer's task, what would encourage them to accept the task, did they gain any benefits from their institution for acting as a reviewer, and how can we improve the reviewing process to make it more appealing?

Most of the reviewers (82%) answered that they had received the review invitation and 86% of respondents think that the time we give them for the review is long enough. For 91% the reviewer's form is written clearly and with enough details. Main reasons for declining the task were: work overload (22%), the paper was out of the reviewer's expertise (14%) and the paper was of poor quality (7%). The answers to the question what would encourage you to accept the task were various: mainly they were related to the paper topic, paper quality, lack of time, impact factor of the journal, and finally to some kind of certification or acknowledgement. Only 19% of reviewers gain any kind of benefit from their institution for acting as a reviewer, which is surprisingly low. As for the suggestions on how to improve the peer reviewing process, there were only a few, such as to check the mailing system (since they had not received the invitation mail), then to improve the FTB Comet interface (our online submission system), give more time to review the paper, and to provide certificate (which is *nota bene* already provided).

As we can see, there is still a lot of work for improvement of the peer review process. Since number of submissions is increasing each year, our job in the future will be even harder. We should consider distributing reviewer's invitations across the globe, involving more early-career researchers in the peer review, and give more credits to the reviewers. Also, the responsibility lies with their mentors and professors to provide guidelines on how to write and review a research paper. Some kind of acknowledgement by the institution would also stimulate researchers to accept the reviewing task. We as editors also need to take part in the task of educating younger generations. Our editorial team holds presentations as part of the course Methodology of scientific work and intellectual property protection at the Faculty of Food Technology and Biotechnology. Also, our newly established Croatian Association for Scholarly Communication (CROASC) is planning to organize workshops on academic writing and to give recommendations to journals regarding, among others, how to conduct and improve the peer review process.

Peer review operates on the goodwill and dedication of scientists and as such is a most noble cause. That is why it must not be taken for granted. We believe that there is room to improve the current situation of reviewer fatigue but responsibility to reduce the pressure on scientists needs to come from everyone involved in the scientific process, starting from educators and mentors, who should teach early-career researchers the importance of writing sound research papers and brushing up the skills of reviewing a research paper, editors who should carefully choose the appropriate number and quality of the papers they send to each reviewer, and most importantly, reviewers' home institutions must find a way of not just recognizing the work of reviewers but also giving them more time for this honourable task.
